# Febrile Respiratory Illness Associated with Human Adenovirus Type 55 in South Korea Military, 2014–2016^1^

**DOI:** 10.3201/eid2306.161848

**Published:** 2017-06

**Authors:** Hongseok Yoo, Se Hun Gu, Jaehun Jung, Dong Hyun Song, Changgyo Yoon, Duck Jin Hong, Eun Young Lee, Woong Seog, Il-Ung Hwang, Daesang Lee, Seong Tae Jeong, Kyungmin Huh

**Affiliations:** Armed Forces Capital Hospital, Seongnam, South Korea (H. Yoo, D.J. Hong, E.Y. Lee, K. Huh);; Agency for Defense Development, Daejeon, South Korea (S.H. Gu, D.H. Song, D. Lee, S.T. Jeong);; Armed Forces Medical Command, Seongnam (J. Jung, C. Yoon, W. Seog, I.-U. Hwang)

**Keywords:** respiratory infections, pneumonia, adenovirus, military personnel, adenovirus, viruses, South Korea

## Abstract

An outbreak of febrile respiratory illness associated with human adenovirus (HAdV) occurred in the South Korea military during the 2014–15 influenza season and thereafter. Molecular typing and phylogenetic analysis of patient samples identified HAdV type 55 as the causative agent. Emergence of this novel HAdV necessitates continued surveillance in military and civilian populations.

Human adenovirus (HAdV) is a common cause of respiratory infections ranging from uncomplicated upper respiratory infections to life-threatening pneumonia. Military personnel, especially new recruits, are predisposed to respiratory infections caused by HAdV (*1*). The substantial effects of HAdV infection in the military have been demonstrated by the marked increase in the incidence of febrile respiratory illness (FRI) in the US military after vaccination against the virus ended in 1999; in turn, the incidence dramatically declined after the vaccine was reintroduced (*2*).

HAdVs are a group of nonenveloped double-stranded DNA viruses comprising 7 species (A–G) and >50 types (*3*). HAdV types belonging to species B (HAdV-3, -7, -11, -16, -21) and E (HAdV-4) are commonly associated with respiratory infections in adults, particularly in military personnel (*4*). Novel types or genomic variants, such as HAdV-14 (*3*) and HAdV-7 (*5*), have been implicated in epidemics of severe infection. HAdV-55, another emerging type reported in China, Turkey, Spain, Singapore, and Israel (*5*,*6*), has been associated with severe clinical manifestations, which often lead to respiratory failure and death (*7*,*8*).

Since fall 2014, we have observed an outbreak of FRI and pneumonia in military personnel in South Korea. In addition to the increased incidence of FRI, patients experienced severe manifestations. We describe the epidemiologic, clinical, and molecular characteristics of FRI in the South Korea military during October 2014–May 2016.

## The Study

We obtained data regarding temporal trends in FRI incidence from military sentinel surveillance, which has been monitoring weekly FRI rates since October 2011. Monthly numbers of patients with pneumonia (inpatients, outpatients, and emergency room patients) were extracted from a computerized data warehouse that stores data from all military hospitals. We identified pneumonia cases by using the International Classification of Diseases and Related Health Problems, 10th Revision, codes J12–J18. The influenza season, which starts in October and ends the following May, was used as a surrogate for the HAdV season in this study. More detaileds information on FRI surveillance is available in the Technical Appendix.

**Figure 1:**
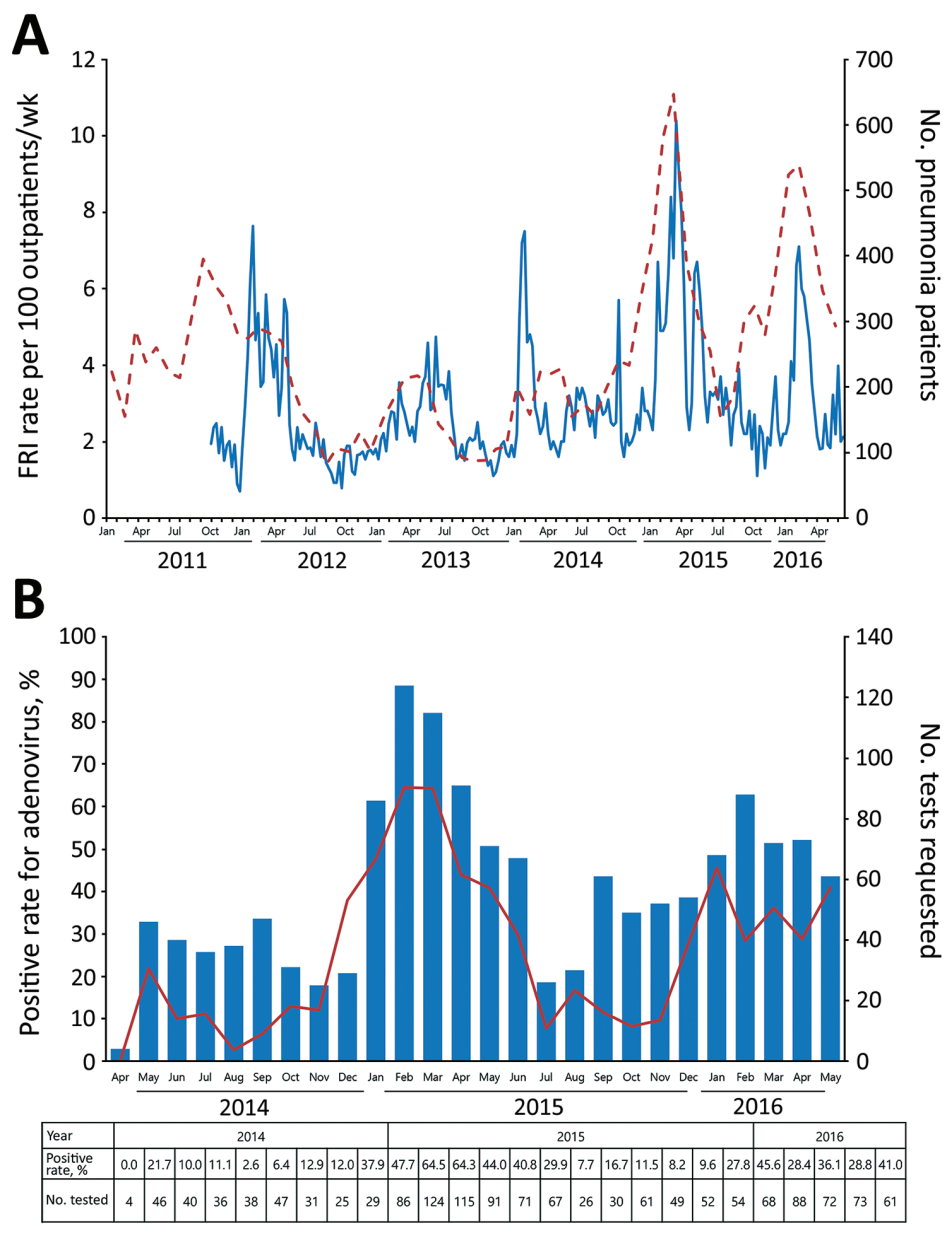
A) Weekly febrile respiratory illness (FRI) rate (solid line) and monthly number of pneumonia patients (dashed line) in the South Korea military, 2011–2016. B) Positive rate of human adenovirus from respiratory specimens (red line) and the number of respiratory virus PCR requested (blue bar) from a tertiary military hospital, South Korea, 2014–2016. The rate and number for each month are shown in the table at bottom.

The trends in FRI rates showed an unusual surge during the 2014–15 influenza season (Figure 1, panel A). The FRI rate increased for 15 weeks in the 2014–15 season, compared with 10 weeks in the 2012–13 season and 5 weeks in the 2013–14 season. Peak FRI rate in the 2014–15 season (10.4%) was higher than rates in the preceding 2 seasons (4.7% and 7.5%). The numbers of pneumonia cases in 2014–15 and 2015–16 seasons were 3,140 and 3,145 patients, respectively, a 191% increase from the mean number during 3 preceding seasons.

In April 2014, a multiplex real-time PCR for identifying 15 viruses from respiratory specimens was introduced at the Armed Forces Capital Hospital, the only tertiary hospital in the South Korea military health care system (detailed methods in Technical Appendix). A total of 1,484 nonduplicate specimens were tested by the end of May 2016 (Figure 1, panel B; Technical Appendix Table 1, Figure). HAdV was identified in 490 (33.0%) of total specimens, and it accounted for 79.7% (282/354) and 53.2% (150/282) of positive results in the 2014–15 and 2015–16 seasons, respectively. Increased HAdV activity was observed from December until the following May.

We reviewed the demographic and clinical information of 878 military patients with FRI or pneumonia who were tested for respiratory viruses from October 2014 through May 2016 (Tables 1, 2). Soldiers of lower rank were markedly more likely to infected with HAdV; soldiers serving in the Air Force were less likely. Patients who had been referred from other hospitals were twice as likely to be HAdV-infected than patients who visited the Armed Forces Capital Hospital directly. Rhinorrhea, sore throat, diarrhea, and nausea/vomiting were more common in patients with HAdV infection. The proportion of patients with pneumonia and the hospitalization rate did not differ between those with and without HAdV infection. However, HAdV-infected patients had a significantly higher risk of requiring intensive care or mechanical ventilator support. In the HAdV-infected group, 8 patients required intubation and 1 died; no one in the noninfected group died or required intubation. Length of hospital stay was also significantly longer among those the HAdV-infected group than among those in the noninfected group (12.6 vs. 9.4 days).

**Table 1:** Epidemiologic characteristics of patients with or without identification of HAdV from respiratory specimens by PCR, South Korea, 2014–2016*

Epidemiologic characteristic	Patients with HAdV, n = 447	Patients with other virus PCR negative for HAdV, n = 431	OR (95% CI)	p value
Year				
Apr 2014–May 2015	274 (65.4)	145 (34.6)	3.13 (2.37–4.12)	<0.001
Jun 2015–May 2016	173 (37.7)	286 (62.3)		
Rank				
Recruit or private	251 (70.5)	105 (29.5)	3.98 (2.98–5.31)	<0.001
PFC or higher	196 (37.5)	326 (62.5)		
Service				
Army	423 (52.1)	391 (47.9)	N\A	<0.001
Navy/Marine Corps	20 (50.0)	20 (50.0)		
Air Force	1 (4.8)	20 (95.2)		
Region				
Seoul/Gyeonggi-do	376 (50.0)	376 (50.0)		0.055
Gangwon-do	46 (65.7)	24 (34.3)		
Chungcheong-do	12 (38.7)	19 (61.3)		
Gyeongsang-do	10 (58.8)	7 (41.2)		
Jeolla-do	3 (37.5)	5 (62.5)		
Route of visit				
Direct	330 (47.3)	367 (52.7)	2.03 (1.45–2.85)	<0.001
Referral	117 (64.6)	64 (35.4)		
Age, y, mean (SD)	20.8 (2.0)	22.2 (5.0)		<0.001

*Values are no. (%) except as indicated. HAdV, human adenovirus; NA, not available; OR, odds ratio; PFC, private first class.

**Table 2:** Clinical characteristics of patients with or without identification of HAdV from respiratory specimens PCR, South Korea, 2014–2016*

Clinical characteristic	Patients with HAdV, n = 447	Patients with other virus or PCR negative for HAdV, n = 431	OR (95% CI)	p value
Presenting symptoms				
Cough†	423 (94.6)	395 (91.9)	1.56 (0.91–2.67)	0.102
Rhinorrhea‡	229 (51.3)	192 (44.7)	1.31 (1.00–1.71)	0.047
Sore throat§	286 (64.3)	207 (48.1)	1.94 (1.48–2.54)	<0.001
Dyspnea¶	60 (13.5)	41 (9.5)	1.48 (0.97–2.25)	0.070
Diarrhea#	125 (33.8)	60 (17.2)	2.46 (1.73–3.49)	<0.001
Nausea/vomiting**	115 (31.0)	58 (16.6)	2.25 (1.58–3.22)	<0.001
Pneumonia	231 (51.7)	250 (58.0)	0.77 (0.59–1.01)	0.060
Hospitalization	277 (62.0)	270 (62.6)	0.97 (0.74–1.28)	0.836
Intensive care	70 (25.3)	30 (11.1)	2.71 (1.70–4.31)	<0.001
Mechanical respiratory support	25 (9.0)	5 (1.9)	5.26 (1.98–13.95)	<0.001
Intubation	8 (2.9)	0	NA	0.005
Death	1 (0.4)	0	NA	0.323
Length of stay, d, mean (SD)	12.6 (9.7)	9.4 (5.0)	NA	<0.001

*Values are no. (%) except as indicated. HAdV, human adenovirus; NA, not available; OR, odds ratio.†n = 877. ‡n = 876.  §n = 875.  ¶n = 876.  #n = 719. **n = 720.

**Figure 2:**
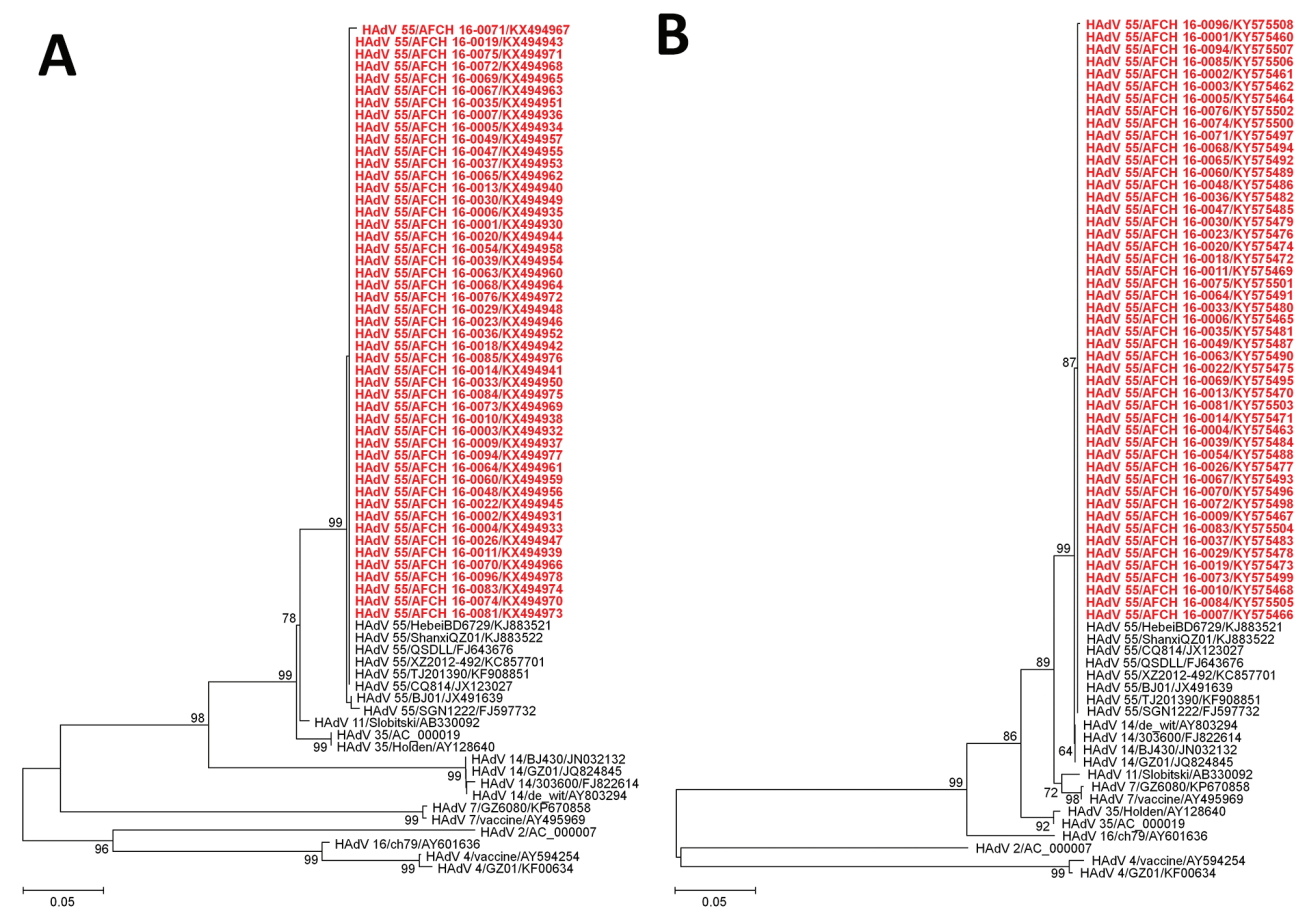
Phylogenetic analysis of human adenoviruses based on the partial nucleotide sequences of hexon (A) and fiber (B) genes, South Korea, 2016. Phylogenetic trees were generated by the neighbor-joining method, using the Kimura 2-parameter method. The percentage of replicate trees in which the associated taxa clustered together in the bootstrap test (1,000 replicates) are shown next to the branches. Red indicates viruses identified in this study. Scale bars denote the number of base substitutions per site.

We conducted molecular typing by the sequencing of hexon and fiber genes with 74 HAdV-positive respiratory specimens collected from March through June 2016 (methods and general characteristics of the patients are available in the Technical Appendix Table 2). Among them, 49 samples were successfully sequenced (GenBank numbers in online Technical Appendix). Phylogenetic analyses showed that all 49 HAdV strains from South Korea clustered with HAdV-55 strains from China, Singapore, Taiwan, Spain, and the United States (Figure 2).

## Conclusions

We describe an outbreak of FRI associated with HAdV in the South Korea military. HAdV is a well-known major cause of FRI in the military, accounting for >50% of FRI and pneumonia cases in military recruits (*1*). Our study also confirmed the predominance of HAdV, which was identified in 49.1% of specimens from patients with FRI or pneumonia. These findings are similar to those of previous studies from South Korea and the United States (*9*,*10*).

The most notable finding of our study is the emergence of HAdV-55 in the South Korea military. HAdV-55 is a novel type that has been associated with a severe clinical course and death in healthy young adults (*7*,*8*). We also found that HAdV infection was associated with intensive care, mechanical ventilator support, and longer hospital stay. In addition, we found that the only patient who died was HAdV infected. From a molecular perspective, HAdV-55 is a novel type with a hexon gene recombination between HAdV-11 and HAdV-14 (*11*). Phylogenetic analysis by using the hexon and fiber gene sequence of 49 strains collected in our study showed that they clustered with previously reported HAdV-55 strains.

Our findings have implications beyond military settings. Spread of infection of traditionally military-associated HAdV types into civilians has been recently reported in the United States and China (*3*,*12*,*13*). Thus, surveillance of HAdV types among both military and civilian populations is warranted; such measures are being implemented by the US Centers for Disease Control and Prevention (Atlanta, GA, USA) (*12*).

Our study has some limitations. First, our findings may not be generalizable due to the retrospective nature of the study. However, the military health system in South Korea provides healthcare exclusively to all military personnel; therefore, epidemiologic information gathered from our surveillance is accurate and comprehensive. Second, we conducted molecular typing with samples collected from February 2016, which was substantially later than the onset of the epidemic. However, HAdV-55 had already been identified in a case series from our center during June 2014–May 2015 (*8*). Because evidence shows that HAdV-55 has been already circulating since early 2014, we believe we can assume that HAdV-55 was the causative agent of the outbreak described in this study. Previously, the HAdV typing study conducted in 2007 reported HAdV-7 as the most prevalent type (*14*). Lack of continuous surveillance makes it difficult to estimate exactly when this novel type was introduced into South Korea.

Further genomic analysis of the collected samples and enhanced surveillance, including of civilian populations, would provide more information on the epidemiology of HAdV infection. In addition, studies are needed on the efficacy of previous vaccines against HAdV-55.

Technical Appendix Detailed methods of the study of febrile respiratory illness associated with human adenovirus in South Korea military, 2014–2016.
