# Supplementary material for: Febrile Respiratory Illness Associated with Human Adenovirus Type 55 in South Korea Military, 2014–2016
Source: Emerg Infect Dis. 2017 Jun;23(6):1016–20. doi: 10.3201/eid2306.161848 (PMC5443441; doi:10.3201/eid2306.161848)
Supplement: Technical Appendix — Detailed methods of the study of febrile respiratory illness associated with human adenovirus in South Korea military, 2014–2016. [file 16-1848-Techapp-s1.pdf]

Article DOI: http://dx.doi.org/10.3201/eid2306.161848

Febrile Respiratory Illness Associated with Human Adenovirus Type 55 in South Korea Military, 2014–2016

Technical Appendix

Methods

Military Surveillance for Febrile Respiratory Illness (FRI)

Military FRI surveillance, activated in October 2011, collects data from 43 walk-in clinics that provide primary care exclusively to military personnel. The sentinel sites were selected to represent all 3 departments of the Armed Forces and various geographic areas of South Korea. FRI was defined as a body temperature ≥38°C and the presence of a cough and/or sore throat. The FRI rate was calculated by dividing the number of FRI patients by the number of total outpatients each week. Each sentinel clinic was required to report their weekly FRI rates using a web-based report system. “Increased activity” was defined as occurring when an FRI rate of a certain week was higher than the upper limit of 95% confidence interval of the pre-season mean FRI rate.

General Characteristics of the Armed Forces Capital Hospital

The Armed Forces Capital Hospital is a 660-bed acute care hospital that is the only tertiary care hospital providing referral service to the entire military health care system in South Korea. It also provides primary and secondary care to the service members stationed in the Seoul/Gyeonggi-do metropolitan area. The military health care system in South Korea provides medical care exclusively to all military service members, except for the small number of patients who choose to use civilian clinics; in that case, they still need to visit a military walk-in clinic first for a referral.

Molecular Analyses for Respiratory Viruses

DNA was extracted from sputum or nasopharyngeal swab specimens by using MagNA Pure LC 2.0 (Roche Diagnostics, Mannheim, Germany). Multiplex real-time PCR was conducted

for 15 respiratory viruses (adenovirus, rhinovirus, influenza viruses A and B, respiratory syncytial viruses A and B, coronaviruses 229E/OC43/NL63/HKU1, parainfluenza viruses 1/2/3, bocavirus, and metapneumovirus) by using Real-Q RV Detection Kit (BioSewoom, Seoul, South Korea) on a LightCycler 480 II (Roche Diagnostics) following the manufacturer's instructions.

Since March 2016, DNA samples from 74 consecutive patients with PCR-confirmed human adenovirus (HAdV) infection were stored at −70°C for further molecular analysis. Stored samples were transported regularly to the Agency for Defense Development for sequencing and genotyping. PCR was performed in 50 μL reactions containing dNTP mixture (2.5 mM), 5 U of LA Taq polymerase (Takara, Shiga, Japan), and 10 μM of each primer. Oligonucleotide primer sequences for 2 sequenced gene were as follows:

Hypervariable Region 7 (HVR7) of the Hexon Gene (*1*)

Hexon gene:

Forward: 5′-CTGATGTACTACAACAGCACTGGCAACATGGG-3′

Reverse: 5′-GCGTTGCGGTGGTGGTTAAATGGGTTTACGTTGTCCAT-3′

Fiber gene:

Forward: 5′-ATGACCAAGAGAGTCCGGCTCAG-3′

Reverse: 5′-ATGCAGTGACTAGGGCTCCAGT-3′

Initial denaturation was conducted at 94°C for 5 min, followed by 15 cycles of denaturation at 94°C for 40 s, annealing at 50°C for 40 s, and elongation at 72°C for 1 min, then followed by 25 cycles of denaturation at 94°C for 40 s, annealing at 52°C for 40 s, and elongation at 72°C for 1 min, in a ProFlex PCR system (Applied Biosystems, Foster City, CA, USA). PCR products were sequenced directly by using an Applied Biosystems 3500 series Genetic Analyzer (Applied Biosystems). GenBank accession numbers for the hexon and fiber genes sequences are KX494930–KX494978 and KY575460–KY575508, respectively. Phylogenetic trees were generated by the neighbor-joining method with 1,000 bootstrap replicates using MEGA 6 (*2*).

Statistical Analysis and Ethical Review

χ^2 or Fisher exact tests were used to compare categorical variables, whereas unpaired Student *t* tests were used to compare continuous variables. A p value <0.05 was considered statistically significant, and all tests were 2-tailed. Statistical analysis was performed by using SPSS Statistics 20 (IBM Corp., Armonk, NY, USA). This study was approved by the Institutional Review Board of AFMC (AFMC-16081-IRB-16–072) with a waiver of informed consent.

References

1. Sarantis H, Johnson G, Brown M, Petric M, Tellier R. Comprehensive detection and serotyping of human adenoviruses by PCR and sequencing. J Clin Microbiol. 2004;42:3963–9. PubMed http://dx.doi.org/10.1128/JCM.42.9.3963-3969.2004

2. Tamura K, Stecher G, Peterson D, Filipski A, Kumar S. MEGA6: Molecular Evolutionary Genetics Analysis version 6.0. Mol Biol Evol. 2013;30:2725–9. PubMed http://dx.doi.org/10.1093/molbev/mst197

Technical Appendix Table 1. Distribution of viruses identified from respiratory specimens during flu seasons 2014–15 and 2015–16, South Korea

	2014–15			2015–16		
Respiratory virus	No. viruses	Proportion among all samples, %	Proportion among positive PCR samples, %	No. viruses	Proportion among all samples, %	Proportion among positive PCR samples, %
Adenovirus	282	49.3	79.7	150	29.1	53.2
Bocavirus	5	0.9	1.4	2	0.4	0.7
Coronavirus 229E	1	0.2	0.3	6	1.2	2.1
Coronavirus NL63/HKU1	1	0.2	0.3	6	1.2	2.1
Coronavirus OC43	9	1.6	2.5	7	1.4	2.5
Influenza virus A	17	3.0	4.8	26	5.0	9.2
Influenza virus B	7	1.2	2.0	26	5.0	9.2
Metapneumovirus	3	0.5	0.8	10	1.9	3.5
Parainfluenza virus 1	0	0	0	3	0.6	1.1
Parainfluenza virus 2	0	0	0	2	0.4	0.7
Parainfluenza virus 3	2	0.3	0.6	2	0.4	0.7
Respiratory syncytial virus A	6	1.0	1.7	0	0	0
Respiratory syncytial virus B	2	0.3	0.6	5	1.0	1.8
Rhinovirus	54	9.4	15.3	57	11.0	20.2
No virus identified	218	38.1		234	45.3	
Total	572	100.0		516	100.0	

Technical Appendix Table 2. Epidemiologic and clinical characteristics of patients from whose respiratory samples adenovirus typing was conducted, South Korea, 2014–2016*

Characteristic	HAdV typing, n = 49 (%)	HAdV overall, n = 447 (%)	p
Epidemiologic			
Rank			
Recruits or privates	29 (59.2)	251 (56.2)	0.762
PFC or higher	20 (40.8)	196 (43.8)	
Service			
Army	45 (91.8)	423 (94.6)	0.298
Navy/Marine Corps	3 (6.1)	20 (4.5)	
Air Force	1 (2.0)	1 (0.2)	
Region			
Seoul/Gyeonggi-do	37 (75.5)	376 (84.1)	0.156
Gangwon-do	9 (18.4)	46 (10.3)	
Chungcheong-do	0	12 (2.7)	
Gyeongsang-do	3 (6.1)	10 (2.2)	
Jeolla-do	0	3 (0.7)	
Route of visit			
Direct	20 (40.8)	330 (73.8)	<0.001
Referral	29 (59.2)	117 (26.2)	
Age, y (mean, SD)	20.4 (1.9)	20.8 (2.0)	0.182
Clinical			
Pneumonia	39 (79.6)	231 (51.7)	<0.001
Hospitalization	46 (93.9)	277 (62.0)	<0.001
Intensive care	17 (37.0)	70 (25.3)	0.002
Ventilatory support	4 (8.7)	25 (9.0)	0.515
Intubation	3 (6.5)	8 (2.9)	0.085
Death	0	1 (0.4)	>0.999

*HAdV, human adenovirus; PFC, private first class.

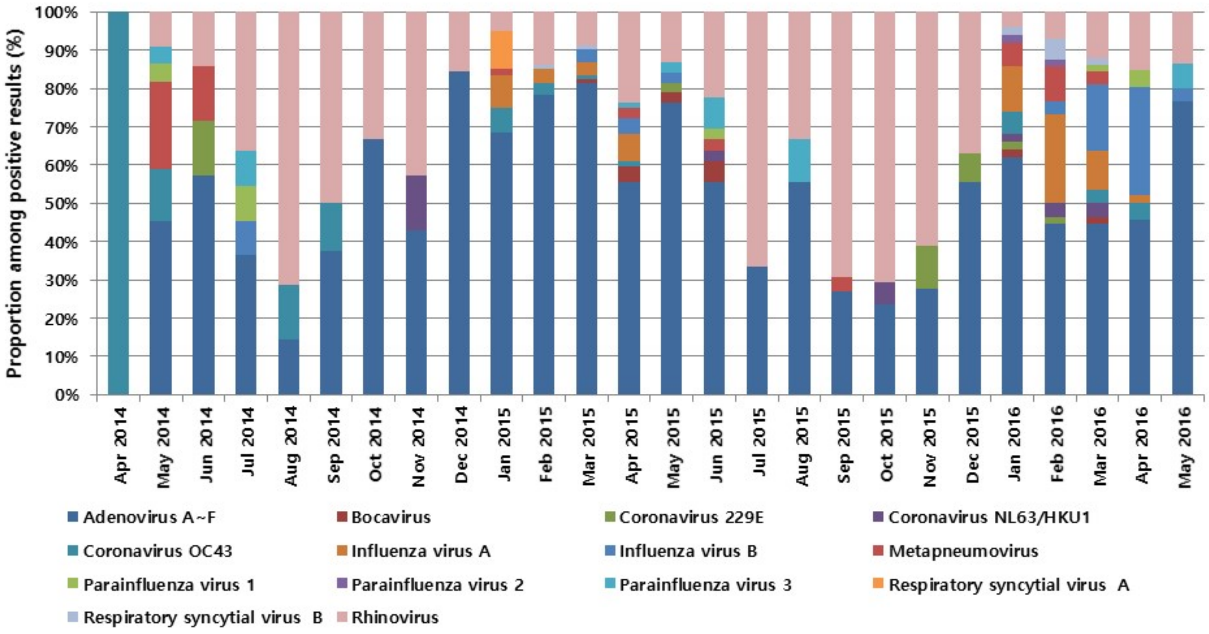

Technical Appendix Figure. Pathogen distribution of respiratory viruses identified by multiplex real-tile PCR at the Armed Forces Capital Hospital, South Korea, 2014–2016.
